# Dropped Head Syndrome As the Initial Presentation of Myasthenia Gravis Leading to Myasthenic Crisis in an Elderly Patient

**DOI:** 10.7759/cureus.114368

**Published:** 2026-08-11

**Authors:** Namrata Maheshwari, Suran Wijethunga, Syeda Ariba Zehra

**Affiliations:** 1 Intensive Care Unit, Medway NHS Foundation Trust, Gillingham, GBR; 2 Critical Care, Medway NHS Foundation Trust, Gillingham, GBR

**Keywords:** bulbar weakness, dropped head syndrome, immunoglobulin therapy, intensive care, myasthenia gravis, myasthenic crisis, neuromuscular weakness, non-invasive ventilation, pyridostigmine, respiratory failure

## Abstract

Myasthenia gravis is an autoimmune disorder of the neuromuscular junction characterized by fatigable, fluctuating skeletal-muscle weakness that classically involves ocular and bulbar muscles, although atypical presentations occur. We describe an 84-year-old man who presented with recurrent falls and acute dropped head syndrome due to predominant neck extensor weakness, without ocular or limb involvement. A clinical diagnosis of myasthenia gravis was made, and during admission he deteriorated rapidly into myasthenic crisis, with bulbar dysfunction and hypercapnic respiratory failure requiring intensive care and mechanical ventilation. Single-fiber electromyography demonstrated markedly increased jitter, supporting the diagnosis; however, myasthenia gravis-specific antibodies were not tested during the acute admission. He was treated with intravenous immunoglobulin, pyridostigmine, and corticosteroids, resulting in clinical improvement and successful extubation. This case highlights dropped head syndrome as a rare initial presentation of myasthenia gravis. It emphasizes the importance of early recognition, as delayed diagnosis may lead to a life-threatening myasthenic crisis.

## Introduction

Myasthenia gravis is an autoimmune disorder of the neuromuscular junction characterized by fatigable, fluctuating skeletal muscle weakness. It is caused by pathogenic antibodies that most commonly target the postsynaptic acetylcholine receptor (AChR, which is present in approximately 80-85% of generalized cases), with antibodies against muscle-specific kinase (MuSK; ~5-8%) and low-density lipoprotein receptor-related protein 4 (LRP4, ~2-3%) accounting for additional cases; around 10% of patients are seronegative on current assays. The condition has a bimodal incidence, with an early-onset, female-predominant peak and a late-onset, male-predominant peak that is increasingly recognized in older adults. It classically presents with fluctuating ocular symptoms such as ptosis and diplopia and may progress to involve bulbar, limb, and respiratory musculature [1,2].

Atypical presentations create significant diagnostic challenges, particularly in late-onset disease (symptom onset at ≥50 years, with very-late-onset disease defined as onset at ≥65 years). The incidence of late-onset myasthenia gravis is increasing, is frequently AChR-positive, and is often associated with anti-titin or anti-ryanodine-receptor antibodies; it is associated with thymoma and tends toward bulbar and axial involvement, with a higher risk of crisis. Dropped head syndrome is characterized by severe weakness of the cervical extensor muscles, producing progressive forward flexion of the neck and an inability to maintain an upright head posture. In an older adult, the differential for dropped head syndrome is broad. It should first consider age-related structural and vascular causes, degenerative or spondylotic cervical spine disease, isolated neck-extensor myopathy, and brainstem (including pontine) ischemia alongside motor neuron disease, inflammatory myopathy, and Parkinsonian antecollis; myasthenia gravis is an uncommon but important cause [3-6]. We present a case of late-onset myasthenia gravis that initially presented with predominant dropped head syndrome and rapidly progressed to myasthenic crisis.

## Case presentation

An 84-year-old man presented to the emergency department with a two-day history of difficulty lifting his chin from his chest, alongside recurrent falls over the preceding three weeks.

The patient had been fully independent until late December 2025. In early January 2026, he underwent permanent pacemaker insertion for symptomatic bradycardia, following admission after a mechanical fall. He experienced several further falls at home that continued despite effective pacing, although he remained able to perform his activities of daily living. Later that month, he was diagnosed with respiratory syncytial virus infection, after which he reported generalized weakness but remained functionally independent. He subsequently presented to the hospital following another fall. CT of the head showed no acute abnormality, and he was discharged, only to be readmitted the following day after a further fall when dropped head syndrome was noted. The recurrent falls were likely multifactorial, reflecting the antecedent symptomatic bradyarrhythmia together with early, evolving axial and proximal weakness superimposed on age-related frailty; notably, they persisted despite pacing.

On examination in the emergency department, he was alert and oriented, with a Glasgow Coma Scale score of 15/15 and normal vital signs. Neurological examination revealed marked neck extensor weakness, with an inability to lift the chin from the chest. There was no ptosis, nystagmus, or other cranial nerve deficit, and upper and lower limb tone, power, and reflexes were clinically normal. Formal Medical Research Council grading and provocative testing for fatigability were not documented at presentation. Respiratory examination showed equal bilateral air entry, with no adventitious breath sounds or signs of respiratory distress.

Approximately 42 hours after admission, he deteriorated rapidly, developing acute hypercapnic (type 2) respiratory failure with reduced consciousness that required emergency airway support. The combination of fatigable neck-extensor weakness and rapidly evolving bulbar and respiratory failure led to a clinical (working) diagnosis of myasthenia gravis, and the deterioration was recognized and managed as a myasthenic crisis. Myasthenia gravis-specific antibodies were not available during the acute admission, and the diagnosis was subsequently supported by neurophysiological findings. He was transferred to the intensive care unit, where arterial blood gas analysis confirmed hypercapnic respiratory failure. At this stage his Glasgow Coma Scale score had fallen to 13/15, and new speech and swallowing difficulties were noted.

Routine hematological and biochemical tests are summarized in Table 1. Most parameters were within normal limits; notable abnormalities included a raised C-reactive protein (35.7 mg/L), a mild normocytic anemia (hemoglobin 121 g/L), and a mildly elevated serum creatinine (115 µmol/L). The raised C-reactive protein was interpreted as an intercurrent inflammatory or infective process, in keeping with the recent respiratory syncytial virus infection and the basal atelectasis seen on imaging, with no clinical evidence of active sepsis. Autoimmune screening, including antinuclear and antineutrophil cytoplasmic antibodies, was negative. Thyroid function tests showed a low free triiodothyronine level, a normal free thyroxine level, and a suppressed thyroid-stimulating hormone, a pattern most consistent with non-thyroidal illness (euthyroid sick) syndrome in the setting of acute illness rather than primary thyrotoxicosis; repeat testing after recovery was advised. The troponin I level was elevated; in the context of critical illness, the recent bradyarrhythmia and pacemaker insertion, and preserved left ventricular function on echocardiography, this was considered most consistent with type 2 (demand) myocardial injury rather than acute coronary occlusion. Myasthenia gravis-specific autoantibodies (AChR, MuSK, and LRP4) were not performed during the acute admission; the diagnosis therefore relied on neurophysiological findings, and serological confirmation was recommended at follow-up.

**Table 1:** Baseline laboratory investigations Investigations performed during admission. The thyroid pattern (low free T3, normal free T4, low TSH) is consistent with non-thyroidal illness (euthyroid sick) syndrome in acute illness rather than primary thyrotoxicosis. The elevated troponin I level was attributed to type 2 (demand) myocardial injury. Serum creatine kinase and myasthenia gravis-specific autoantibodies were not performed. TSH: thyroid-stimulating hormone, ANA: antinuclear antibody, ANCA: antineutrophil cytoplasmic antibody, T3: triiodothyronine, T4: thyroxine

Parameter	Patient value	Reference range
White blood cell count	6 × 10⁹/L	4.0-11.0 × 10⁹/L
Haemoglobin	121 g/L	130-180 g/L
Platelet count	181 × 10⁹/L	150-400 × 10⁹/L
C-reactive protein	35.7 mg/L	<5 mg/L
Sodium	142 mmol/L	135-145 mmol/L
Potassium	4.7 mmol/L	3.5-5.0 mmol/L
Calcium	2.23 mmol/L	2.15-2.60 mmol/L
Magnesium	0.77 mmol/L	0.70-1.00 mmol/L
Phosphate	1.17 mmol/L	0.80-1.50 mmol/L
Serum creatinine	115 µmol/L	60-110 µmol/L
Troponin I	245.9 ng/L	<34 ng/L
Free T3	2.9 pmol/L	3.1-6.8 pmol/L
Free T4	18.6 pmol/L	12-22 pmol/L
TSH	0.04 mIU/L	0.27-4.20 mIU/L
ANA	Negative	Negative
ANCA	Negative	Negative
Neuronal antibodies	Negative	Negative
Infectious/sexual screening	Negative	Negative

MRI of the brain and cervical spine was deferred because of the recently implanted pacemaker, in keeping with local policy for newly implanted devices; the precise MR-conditional status of the device was not recorded. Imaging and ancillary investigations are summarized in Table 2. CT of the head and cervical spine showed no acute intracranial abnormality and no structural cause for the dropped head; in particular, no fracture or destructive osseous lesion was identified. CT of the chest, abdomen, and pelvis showed basal atelectasis but no evidence of malignancy, and no thymic mass was reported; however, this was a standard staging study rather than a dedicated thymic protocol. Given the recognized association between myasthenia gravis and thymic pathology, dedicated thymic imaging (contrast-enhanced CT or MRI of the mediastinum) is planned at outpatient follow-up to exclude thymoma or thymic hyperplasia. Echocardiography, performed to evaluate the troponin rise and bradyarrhythmia, showed preserved left ventricular systolic function with an ejection fraction greater than 50%.

**Table 2:** Imaging and ancillary investigations MRI was deferred because of the recently implanted pacemaker. CT: computed tomography, EF: ejection fraction, MRI: magnetic resonance imaging

Investigation	Finding
MRI of brain and cervical spine	Not performed because of recent permanent pacemaker insertion
CT of head	No acute intracranial abnormality
CT of cervical spine	No fracture or destructive osseous lesion; no structural cause for the dropped head identified
CT of chest, abdomen, and pelvis	Basal atelectasis; no evidence of malignancy and no thymic mass reported (standard staging study, not a dedicated thymic protocol)
Echocardiography	Preserved left ventricular systolic function with ejection fraction greater than 50%
Spirometry	Performed during admission; the available report suggested a possible restrictive pattern (quantitative values not recorded)

**Figure 1:**
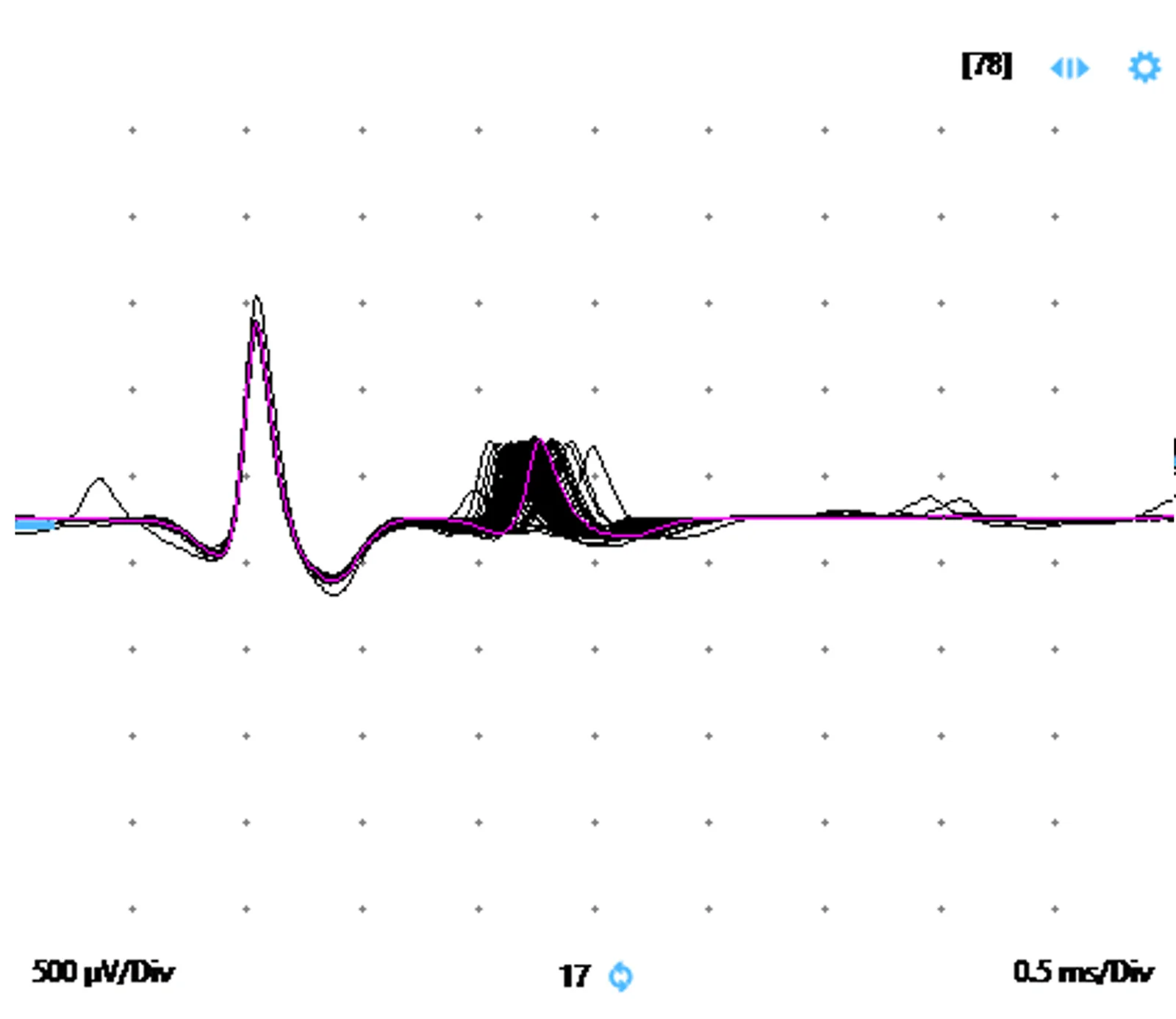
Single-fiber electromyography of the left orbicularis oculi demonstrating markedly increased jitter Superimposed consecutive discharges of a muscle-fiber pair recorded from the left orbicularis oculi (gain 500 µV/division; sweep 0.5 ms/division). The action potential of the triggering fiber (left) is tightly superimposed. In contrast, the second potential (right) varies widely in latency between consecutive discharges, indicating markedly increased jitter and impaired neuromuscular-junction transmission.

**Figure 2:**
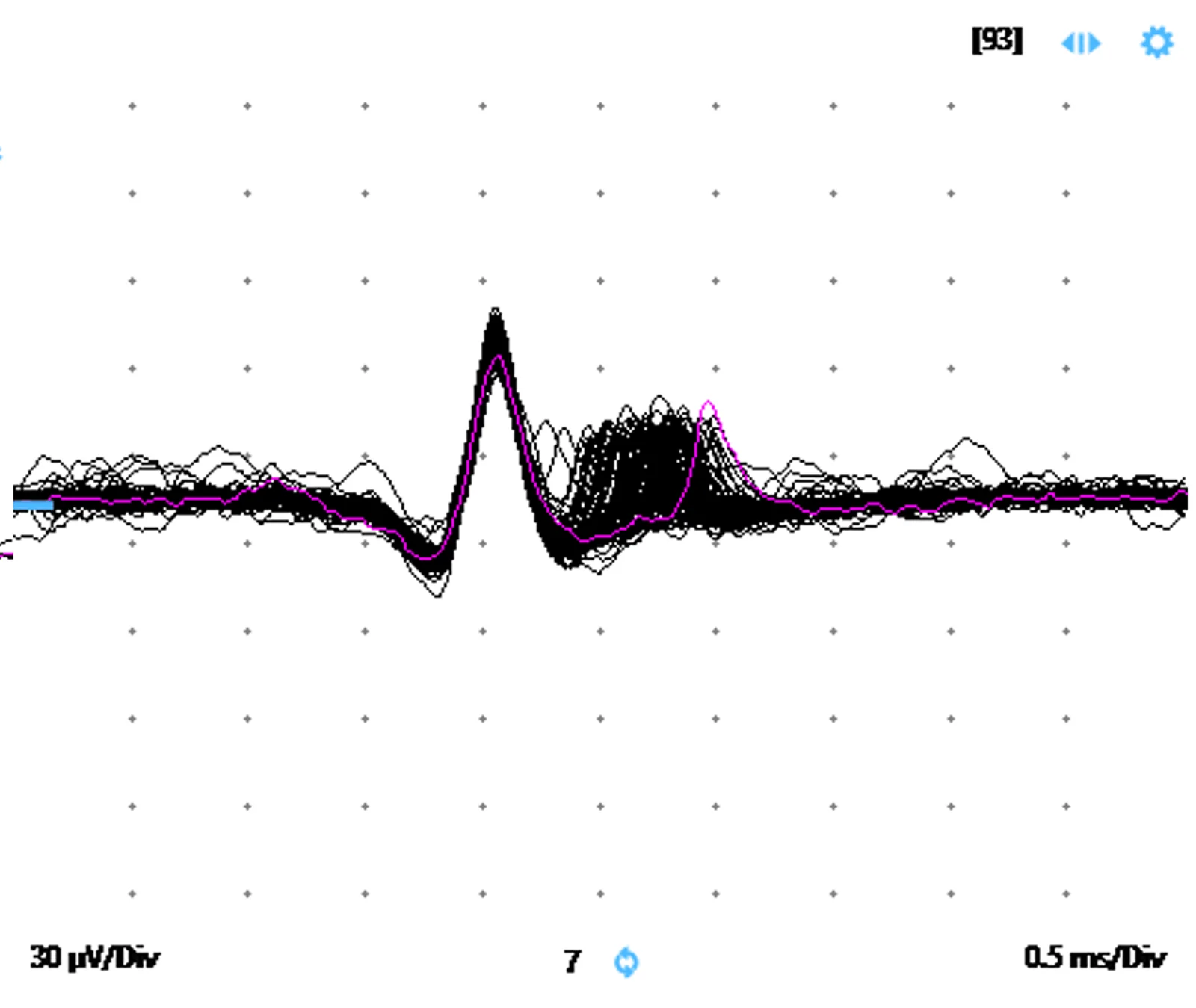
Second single-fiber electromyography recording from the left orbicularis oculi A second representative muscle-fiber pair from the left orbicularis oculi (gain 30 µV/division; sweep 0.5 ms/division), again showing a stable triggering potential with marked latency variability of the second potential across consecutive discharges, consistent with increased jitter. Recordings were obtained despite pyridostigmine being administered approximately six hours before testing, which tends to reduce jitter.

Neurophysiological testing was performed during the admission to differentiate a neuromuscular-junction disorder from a Guillain-Barré syndrome variant, given the acute bulbar symptoms and neck weakness; the findings are summarized in Table 3. Nerve conduction studies of the left upper limb (median and ulnar motor, radial sensory) showed mild slowing of motor conduction velocities in keeping with age, without conduction block or significantly prolonged F-wave latencies, and no evidence of a demyelinating neuropathy. Needle electromyography of the right first dorsal interosseous, left biceps, and left deltoid showed no fibrillations, positive sharp waves, or fasciculations, with normal motor-unit morphology and full recruitment; the cervical paraspinal extensors were not sampled. Repetitive nerve stimulation at 3 Hz of the left abductor digiti minimi and left upper trapezius, with the latter being an axial muscle innervated by the spinal accessory nerve, showed baseline first-to-fifth amplitude decrements of -1.8% and -2.0%, respectively, below the ≥10% threshold for abnormality. Single-fiber electromyography of the left orbicularis oculi was markedly abnormal, with a mean jitter of 106.9 µs and 10 of 18 potential pairs abnormal, despite the administration of pyridostigmine approximately six hours before testing. This factor tends to reduce jitter, so an abnormal result under these conditions strengthens the finding (Figures 1-2). The combination of a non-significant decrement on repetitive nerve stimulation with markedly increased jitter on single-fiber electromyography is well recognized, as single-fiber electromyography is the most sensitive electrodiagnostic test for neuromuscular-junction dysfunction. The overall findings indicated impaired neuromuscular-junction transmission, favoring myasthenia gravis over a Guillain-Barré variant.

**Table 3:** Neurophysiological studies Neurophysiological studies performed during admission EMG: electromyography, RNS: repetitive nerve stimulation, SFEMG: single-fiber electromyography

Investigation	Finding
Nerve conduction studies	Left median and ulnar motor and left radial sensory studies: mild slowing of motor conduction velocities (median 45 m/s, ulnar 47 m/s) without conduction block or significantly prolonged F-wave latencies, in keeping with age; no evidence of a demyelinating neuropathy
Needle electromyography (right first dorsal interosseous, left biceps, left deltoid)	No fibrillations, positive sharp waves or fasciculations; normal motor-unit amplitude, duration, and recruitment with full interference patterns; no evidence of denervation or myopathy. The cervical paraspinal extensors were not sampled
Repetitive nerve stimulation (3 Hz, left abductor digiti minimi and left upper trapezius)	Baseline first-to-fifth amplitude decrement of -1.8% (abductor digiti minimi) and -2.0% (upper trapezius), below the ≥10% abnormality threshold; corresponding area decrements -7.9% and -12.3%; post-exercise responses were not documented in the report
Single-fiber electromyography	Left orbicularis oculi: markedly increased jitter, with a mean jitter of 106.9 µs and 10 of 18 potential pairs abnormal, despite pyridostigmine approximately six hours before testing (a factor that tends to reduce jitter); impulse blocking was not separately quantified
Overall interpretation	Findings favored myasthenia gravis over a Guillain-Barré syndrome variant: markedly abnormal single-fiber electromyography indicating impaired neuromuscular-junction transmission, with no evidence of demyelinating neuropathy or denervation

The initial differential diagnosis was broad. Structural cervical spine pathology, including cervical myelopathy or traumatic injury, was considered but was not supported by CT imaging. Acute ischemic stroke was considered; however, a normal non-contrast CT head does not exclude acute ischemia, particularly in the posterior fossa or brainstem, and stroke was felt to be less likely given the selective, fatigable pattern of weakness and the subsequent neurophysiological findings, recognizing that MRI, the more sensitive investigation, was precluded by the pacemaker. Guillain-Barré syndrome was also considered given the acute bulbar and neck weakness; however, nerve conduction studies showed no evidence of a demyelinating neuropathy, making this unlikely. Motor neuron disease can present with dropped head syndrome, but the acute respiratory deterioration without widespread upper or lower motor neuron signs was atypical. Inflammatory myopathy was considered less likely on clinical and neurophysiological grounds, although it could not be excluded biochemically because a serum creatine kinase level was not available. Parkinsonian disorders were also considered. The subsequent rapid development of bulbar symptoms and hypercapnic respiratory failure strongly suggested a neuromuscular junction disorder, supported by neurophysiology.

Management followed the standard approach to myasthenic crisis, combining intensive-care ventilatory support with a rapid-acting immunotherapy and corticosteroids. On admission to the intensive care unit, he initially received non-invasive ventilation with bilevel positive airway pressure. However, he required endotracheal intubation the following day because of progressive respiratory muscle fatigue and worsening hypercapnia. Rapid-acting immunotherapy was given as a five-day course of intravenous immunoglobulin (the standard total dose is 2 g/kg over five days; the patient-specific weight-based dose was not recorded in the notes available). Corticosteroid therapy was initiated cautiously with prednisolone 10 mg on alternate days and escalated gradually during intravenous immunoglobulin therapy, a strategy chosen deliberately to avoid the recognized risk of steroid-induced early exacerbation in a patient with bulbar and respiratory involvement. Pyridostigmine was used at 30 mg three times daily and, following neurophysiological assessment, increased to 60 mg three times daily. As a matter of standard practice, acetylcholinesterase inhibitors are withheld in intubated patients during myasthenic crisis to reduce bronchial secretions and avoid a cholinergic contribution to weakness that cannot readily be distinguished from myasthenic weakness in a ventilated patient, and are reintroduced as strength recovers, usually around extubation.

His ICU course was complicated by unplanned self-extubation, which required urgent re-intubation. However, following completion of intravenous immunoglobulin and optimization of oral myasthenia gravis therapy, his bulbar and respiratory muscle strength improved significantly, and he was successfully extubated. After a period of intensive physiotherapy and rehabilitation, he was stepped down from high-dependency care and subsequently discharged from the respiratory ward.

At discharge, he was alert and fully oriented, mobilizing with a walking frame and the assistance of one person. Neurological examination demonstrated global muscle strength of 4/5 with no focal neurological deficit, and his National Early Warning Score 2 (NEWS2) was 0 [7]. The change from the initially normal limb power recorded at presentation to mild global weakness at discharge was attributed to generalization of myasthenia gravis together with intensive-care-acquired weakness and deconditioning after prolonged ventilation; a discrete discharge neck-extensor grade was not separately documented. Although he had made significant neurological recovery, he remained at increased risk of falls, and therapy input focused on mobility, transfers, and safety awareness.

He was discharged home with a home care package providing three visits daily and community therapy input, with a referral to the falls prevention team. His discharge medications for myasthenia gravis comprised pyridostigmine 60 mg three times daily, prednisolone on a gradually escalating regimen, and lansoprazole for gastric protection. Neurology outpatient follow-up was arranged, including a plan to perform a myasthenia gravis autoantibody panel (AChR, MuSK, and LRP4 antibodies, together with anti-titin and anti-striational antibodies). Because intravenous immunoglobulin infuses large quantities of pooled donor immunoglobulin that can confound autoantibody assays, producing false-positive results or altered titers, this serological testing was deliberately deferred until the infused immunoglobulin had adequately cleared; given the approximately three- to four-week half-life of infused IgG, an interval of several weeks to a few months is appropriate before samples for antibody testing are obtained and the results interpreted. Dedicated thymic imaging was also planned. His general practitioner was asked to monitor steroid titration, blood pressure, and glycemic control, with repeat thyroid function testing advised.

For the ceiling of care, a do-not-attempt cardiopulmonary resuscitation decision was agreed upon during admission following discussion with the patient and his family. The agreed ceiling of care was selective treatment without reintubation.

## Discussion

Dropped head syndrome is an uncommon manifestation of myasthenia gravis and represents a diagnostic pitfall, particularly in older adults. Myasthenia gravis typically presents with ocular symptoms such as ptosis and diplopia. In contrast, isolated axial weakness may be misattributed to frailty, degenerative cervical spine disease, motor neuron disease, inflammatory myopathy, or Parkinsonian syndromes [5,6,8]. This case demonstrates that the absence of classic ocular symptoms does not exclude the diagnosis.

Selective weakness of the cervical extensor muscles creates a mechanical imbalance, producing progressive forward flexion of the neck and difficulty maintaining an upright head posture. In this patient, the recurrent falls may have represented an early manifestation of evolving axial weakness before the development of overt dropped head syndrome. The acute onset and subsequent rapid progression to bulbar dysfunction and hypercapnic respiratory failure were important clues pointing to a neuromuscular junction disorder rather than structural cervical spine disease or a central neurological cause.

The patient’s recent respiratory syncytial virus infection probably contributed to the deterioration. Infection is a well-recognized precipitant of myasthenic crisis and may unmask previously undiagnosed neuromuscular junction disease [2,9]; in this case, it preceded rapid progression from predominant neck extensor weakness to life-threatening respiratory failure requiring mechanical ventilation.

Dropped head syndrome as the presenting feature of myasthenia gravis is rare but not unprecedented. Early reports drew attention to the sign [3,4], and subsequent series have established head drop as a recognized, prognostically relevant axial phenotype, particularly in late-onset disease: Sih et al. found head drop to be a frequent feature of late-onset myasthenia gravis, and Tamai et al. reviewed its presentation and treatment [5,6]. Our patient is consistent with these cohorts in his older age and axial-predominant onset but is notable for dropped head syndrome that was the predominant initial feature, occurred without ocular involvement, and progressed within days to myasthenic crisis. This trajectory underscores the danger of attributing such weakness to frailty or degenerative disease in an elderly faller.

This patient’s antibody status was not determined, and the serological subtype therefore cannot be assigned. The ocular-sparing pattern of bulbar, neck, and respiratory weakness shows some phenotypic overlap with MuSK myasthenia gravis, which has a predilection for these muscle groups, frequently spares the ocular muscles, carries a higher risk of crisis, often responds poorly to acetylcholinesterase inhibitors, and tends to respond to rituximab. However, MuSK-associated disease is typically female-predominant with a younger age at onset. In contrast, this patient, an elderly man with very-late-onset disease, fits far better demographically with AChR-positive late-onset myasthenia gravis, which is frequently associated with anti-titin and anti-ryanodine-receptor antibodies. The subtype cannot be determined from the phenotype alone and requires serological testing, which was recommended at follow-up.

Severe dropped head syndrome can itself compromise the airway and ventilation. Fixed forward flexion of the neck may cause upper-airway obstruction and mechanically restrict chest-wall expansion, compounding the diaphragmatic and bulbar weakness of a myasthenic crisis and accelerating hypercapnic respiratory failure. This mechanical contribution may have hastened the respiratory decompensation observed in this patient.

This case also highlights the importance of maintaining diagnostic suspicion when investigations are limited. The recent pacemaker implantation precluded MRI under the local policy, and myasthenia gravis-specific serology was not performed acutely, so neurophysiology played the central diagnostic role; nonetheless, treatment should not be delayed when the clinical picture suggests a myasthenic crisis. In intubated patients in myasthenic crisis, withholding acetylcholinesterase inhibitors is the recommended standard approach rather than a matter of debate. These agents increase bronchial secretions and can produce a cholinergic contribution to weakness that is difficult to distinguish from myasthenic weakness in a ventilated patient, so they are held during mechanical ventilation and reintroduced as strength recovers, usually around extubation [9].

Early recognition and escalation were critical in this case. Intensive care support, intravenous immunoglobulin, pyridostigmine, corticosteroids, and multidisciplinary rehabilitation were associated with meaningful short-term neurological and respiratory recovery. The key learning points are that dropped head syndrome can be a rare initial presentation of myasthenia gravis, that the absence of ocular symptoms does not exclude the diagnosis, that unexplained falls with acute neck weakness should prompt evaluation for a neuromuscular junction disorder, and that recent infection may precipitate rapid progression to myasthenic crisis.

This report has limitations. In this retrospective single-patient case report, myasthenia gravis-specific serology (AChR, MuSK, and LRP4 antibodies) was not performed during the acute admission, and the diagnosis therefore rested on neurophysiology and is best regarded as supported rather than definitively confirmed; the serological subtype remains undetermined. Standardized Medical Research Council grading, documented provocative fatigability testing, serial bedside respiratory parameters (forced vital capacity or negative inspiratory force), the arterial blood gas values at decompensation, and the serum creatine kinase were not available in the records used for this report, and a full medication review was not documented. Confirmatory serology and thymic evaluation are recommended and are being sought at outpatient follow-up.

## Conclusions

Dropped head syndrome is a rare but important initial manifestation of myasthenia gravis, particularly in older adults. It may occur even in the absence of typical ocular features such as ptosis or diplopia. In patients presenting with acute neck extensor weakness, recurrent unexplained falls, bulbar symptoms, or evolving respiratory compromise, clinicians should consider a neuromuscular junction disorder early in the differential diagnosis.

In this patient, clinically suspected myasthenia gravis, strongly supported by neurophysiological findings, progressed rapidly to myasthenic crisis. The central message is simple: consider myasthenia gravis in any older adult with acute neck extensor weakness or unexplained falls, monitor respiratory function closely, and treat early to prevent life-threatening respiratory failure.
